# Chronic Pain Following Physical and Emotional Trauma: The Station Nightclub Fire

**DOI:** 10.3389/fneur.2014.00086

**Published:** 2014-06-02

**Authors:** Rachel Egyhazi, Felipe Fregni, Gabriela L. Bravo, Nhi-Ha T. Trinh, Colleen M. Ryan, Jeffrey C. Schneider

**Affiliations:** ^1^Department of Physical Medicine and Rehabilitation, Spaulding Rehabilitation Hospital, Harvard Medical School, Boston, MA, USA; ^2^Neuromodulation Center, Spaulding Rehabilitation Hospital, Harvard Medical School, Boston, MA, USA; ^3^Depression and Clinical Research Program, Department of Psychiatry, Massachusetts General Hospital, Boston, MA, USA; ^4^Department of Surgery, Sumner Redstone Burn Center, Massachusetts General Hospital, Boston, MA, USA; ^5^Shriners Hospitals for Children-Boston, Boston, MA, USA

**Keywords:** fire, burn, graft, pain, depression

## Abstract

**Objective:** The purpose of this study was to evaluate factors associated with chronic pain in survivors of a large fire, including those with and without burn injury.

**Methods:** This study employed a survey-based cross-sectional design to evaluate data from survivors of The Station nightclub fire. The primary outcome measure was the presence and severity of pain. Multiple linear regressions with a stepwise approach were used to examine relationships among variables. Variables considered included age, gender, marital status, burn injury, total body surface area, skin graft, pre-morbid employment, time off work, return to same employment, depression (Beck depression inventory, BDI), and post-traumatic stress (impact of event scale – revised).

**Results:** Of 104 fire survivors, 27% reported pain at least 28 months after the event. Multiple factors associated with pain were assessed in the univariate analysis but only age (*p* = 0.012), graft (*p* = 0.009), and BDI score (*p* < 0.001) were significantly associated with pain in the multiple regression model.

**Discussion:** A significant number of fire survivors with and without burn injuries experienced chronic pain. Depth of burn and depression were significantly associated with pain outcome. Pain management should address both physical and emotional risk factors in this population.

## Introduction

Burns are associated with a significant amount of pain during the course of acute care and rehabilitation. Pain has been reported up to 7–12 years after initial injury for a subset of burn patients (1–3). To date, there is limited data regarding the prevalence and characteristics of chronic pain in burn patients, and almost no information regarding pain in fire survivors without physical injury.

Chronic pain is a complex phenomenon involving sensory, physiologic, and cognitive–behavioral components. Chronic pain states are thought to be secondary to molecular and structural reorganization that occur as a result of changes in the sensitivity of the nociceptor peripheral terminal (peripheral sensitization), as well as augmentation of nociceptive synaptic transmission in the dorsal horn of the spinal cord and brain-related pain processing areas such as thalamus, cingulate gyrus, and insula (central sensitization) (4–6). Such mechanisms contribute to altered pain thresholds; pain may be experienced clinically as sensory abnormalities including hyperalgesia, hyperpathia, or allodynia (5, 6).

Psychological factors are closely associated with the development and perpetuation of chronic pain states. Emotional experiences can induce neurophysiological changes in important modulating areas of chronic pain, such as the primary motor cortex. It has been recently shown that catastrophizing thoughts are associated with changes in corticospinal excitability in patients with chronic pain (7).

Chronic pain in fire survivors presents a unique challenge to medical practitioners in that we have yet to fully understand the physiological and psychosocial factors that contribute to pain phenomena in this population. Therefore, research that includes long-term follow-up of burn survivors is imperative since medical literature reports that although pain due to burn injury is a well-described clinical finding and one of the highest research priorities in the field, it remains undertreated (8). The recognition of factors that contribute to chronic pain can provide a foundation, which medical practitioners can use to guide their treatments.

Experiences of chronic pain appear to be variable among burn survivors, and range from discomfort at the scar site, to focal or generalized neuropathic-like pain and altered sensation (2, 8, 9). Fire survivors are often subject to significant emotional trauma related to the event itself (10). It is estimated that 20–45% of burn survivors experience post-traumatic stress disorder (PTSD), associated with delusional memories, nightmares, and pain (11). Schneider et al. demonstrated emotional trauma to be more significant than physical injury in predicting long-term impairments in quality of life for survivors of The Station nightclub fire (12).

The Station nightclub of West Warwick, Rhode Island caught fire during an indoor rock concert on February 20, 2003, after pyrotechnic sparks ignited the stage. Of the estimated 462 people in attendance, 100 people died and 230 were injured from burns, smoke inhalation, and trampling (13). One of the deadliest fires in American history, this catastrophic event provides a unique platform to evaluate relationships between pain, physical trauma, and emotional impairment. The objective of this study was to evaluate factors associated with chronic pain in survivors of a large fire.

## Materials and Methods

### Participants

We employed a cross-sectional study design to evaluate data from survivors of The Station nightclub fire. All persons in attendance at The Station nightclub on February 20, 2003 were eligible for inclusion. There were no explicit exclusion criteria. The Partners Human Research Committee approved all procedures utilized in this study.

### Procedures: Recruitment and survey design

Subjects were recruited from June 2005 to October 2007. Participants were asked to complete a survey consisting of 130 questions pertaining to demographic, medical, psychological, social, and occupational status. Details regarding the full recruitment process and questionnaire are presented in Schneider et al. (12).

### Measures

The primary outcome measure in this dataset analysis was the presence of pain, as assessed by the visual analog scale (VAS). All subjects were asked to assess their level of pain using a horizontal axis scaled from 0 to 10, corresponding to “no pain,” and “worst pain imaginable,” respectively.

Independent variables selected from the survey included demographic, medical, and psychosocial characteristics. Demographic characteristics included age, gender, and marital status. Medical characteristics included the presence of burn (yes/no), as well as the severity of burn, measured by percentage of total body surface area (TBSA: 0, 1–20, ≥21%), skin grafting (yes/no), and medications used. Psychosocial characteristics included employment and psychological impairment. Specifically, participants were asked if they were employed or in school prior to the fire, duration of time off from work or school as a result of the fire, and whether or not they were able to return to the same occupation after the fire. Degree of psychological impairment was assessed using the Beck depression inventory (BDI) and impact of event scale – revised (IES-R) to evaluate for symptoms of depression and post-traumatic stress, respectively.

### Data analysis

Statistical analyses were performed using STATA/IC 12 (StataCorp LP, TX, USA). The first step of modeling involved the selection of covariates. Univariate analysis was performed for each of the predictors using linear regression with only one variable, from which values for the unadjusted β coefficients, *p* values, and 95% confidence intervals (CIs) were obtained. Next, a stepwise multiple regression with a manual backward elimination approach was used to arrive at the most parsimonious model; all variables with a *p* value <0.05 in the univariate analyses were included initially. During the modeling process, the variable with the highest *p* value was eliminated from the maximum model, conditioned on the *p* value being larger than the pre-determined level of 0.05. After evaluating the fit of the reduced model, the next variable with the highest non-significant *p* value was removed. This process was continued until no additional variables could be eliminated from the model. In order to avoid excluding non-significant confounders, each of the excluded variables were examined by adding them individually to the model; confounding was defined by changes of ≥10% in the β coefficient. Any variable identified as an important confounder or clinically relevant was forced into the final model.

As a final consideration, an interaction term was constructed for each paired combination of significant variables from the final multiple regression model. This identified whether or not the simultaneous influence of the two variables on the remaining variable was additive. An additional regression analysis was performed using an interaction term created from all of the three significant predictors to evaluate higher order interactions.

## Results

### Characteristics of the study sample

Of the estimated 462 people in attendance at The Station nightclub at the time of the fire, 362 people survived (12, 13). The recruitment process identified 144 eligible participants, of which 104 people completed the survey.

Demographic, medical, and psychosocial characteristics of respondents are presented in Table 1. At the time of the fire, survivors ranged in age from 19 to 57 years; the mean age was 32 and the mode was 39 years. Almost one-half (47%) experienced a burn injury, of which the most common size was ≤20% TBSA (59%), followed by 21–40% TBSA (27%). The most common areas burned included the head (75%) and upper extremities (65%). Twenty-nine percent of people who sustained a burn injury received a graft. Nearly all participants were found to exhibit some degree of depressive symptoms, with a minority of survivors (22%) reporting moderate to severe symptoms. Nearly one-half (49%) of survivors were identified as having moderate to severe symptoms of post-traumatic stress. Survey of employment status demonstrated that 34% of survivors returned to full-time work after the fire, with 80% returning to their pre-morbid occupation. Time off from work was variable, ranging from a leave of absence of 1 week (30%) to more than 1 year (16%).

**Table 1:** **Characteristics of study sample**.

Category	Characteristics	Number (%), *n* = 104^a^
Demographic	Gender
	Female	40 (38%)
	Age at time of fire, mean years (SD)	32.4 (7.2)
	Marital status
	Married or long-term partner	94 (90%)
Medical	Survivors with burn injury	49 (47%)
	Total body surface area burned
	≤20%	29 (59%, *n* = 49)
	21–40%	13 (27%, *n* = 49)
	>40%	7 (14%, *n* = 49)
	Graft	14 (29%, *n* = 49)
Psychiatric	Depressive symptoms (BDI)^b^
	Minimal	66 (63%)
	Mild	9 (9%)
	Moderate	14 (13%)
	Severe	9 (9%)
	Post-traumatic stress symptoms (IES-R)^c^
	Subclinical	22 (21%)
	Mild	29 (28%)
	Moderate	23 (22%)
	Severe	28 (27%)
Employment	Pre-morbid employment
	Full-time	83 (80%)
	Part-time	9 (9%)
	Student	5 (5%)
	Unemployed	3 (3%)
	Not specified	4 (4%)
	Employment after fire
	Full-time	35 (34%)
	Part-time	9 (9%)
	Unemployed	12 (12%)
	Not specified	48 (46%)
	Return to same job post-fire	83 (80%)
	Time off work
	1–7 days	31 (30%)
	1–3 weeks	17 (16%)
	1–5 months	18 (17%)
	6–12 months	12 (12%)
	>1 year	17 (16%)

*^a^Percentages are relative to total number of study participants (*n* = 104), unless otherwise indicated*.

*^b^BDI, Beck depression inventory*.

*^c^IES-R, impact event scale – revised*.

**Figure 1:**
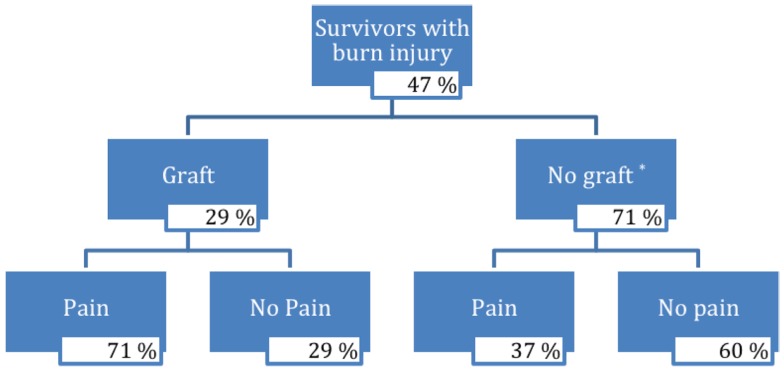
**Flow chart of survivors with burn injury**. *Three percent of burn injury survivors who did not receive a graft did not respond.

Pain characteristics of the study sample are demonstrated in Table 2 and Figure 1. Of the 104 participants surveyed, 27 people (27%) reported pain (VAS from 1 to 10); the average pain score was 3.1 ± 2.6 (mean ± SD). Pain among subgroups of patients with varying burn characteristics was further analyzed. Of those 14 survivors who underwent grafting due to burn, 71% reported pain at the time of the study. This was nearly double the percentage of burn survivors without grafts who experienced pain (37%). Of those grafted patients with pain, the mean VAS pain score was 3.4 ± 2.9, with an average VAS score of 2.4 ± 2.9 for all grafted survivors. Non-grafted burn patients with pain were found to have a mean VAS pain score of 3.0 ± 2.5, with an average VAS score of 1.1 ± 2.1 for this group overall. Only 4 of 50 participants without burn reported pain, with an average VAS score of 3.0 ± 2.8; of these, 1 patient was noted to have sustained a crush injury. The mean VAS pain score for all survivors without burn was 0.2 ± 1.1.

**Table 2:** **Pain characteristics of study sample**.

Characteristic	Number (%)
Fire survivors	104
Unknown level of pain	3
Pain, VAS 1–10	27 (27%)
No pain, VAS 0	74 (73%)
Mean VAS, all fire survivors with pain (SD)	3.1 (2.6)
Burn with graft	14 (29%)
Pain, VAS 1–10	10 (71%)
No pain, VAS 0	4 (29%)
Mean VAS, grafted survivors with pain (SD)	3.4 (2.9)
Mean VAS, all grafted survivors (SD)	2.4 (2.9)
Burn without graft	35 (71%)
Pain, VAS 1–10	13 (37%)
No pain, VAS 0	21 (60%)
No response^a^	1 (3%)
Mean VAS, non-grafted survivors with pain (SD)	3.0 (2.5)
Mean VAS, all non-grafted survivors (SD)	1.1 (2.1)
No burn	50 (51%)
Pain, VAS 1–10	4 (8%)
No pain, VAS 0	28 (56%)
No response^a^	18 (36%)
Mean VAS, non-burn survivors with pain (SD)	3.0 (2.8)
Mean VAS, all non-burn survivors (SD)	0.2 (1.1)

*^a^Considered equivalent to VAS 0 for purposes of mean calculation*.

### Univariate and multivariate analysis

The results of the univariate analysis are presented in Table 3. The variables that were significantly associated with VAS pain score included: age (*p* = 0.009), burn injury (*p* = 0.012), graft (*p* = 0.006), time off work (*p* = 0.001), return to same employment (*p* < 0.001), BDI score (*p* < 0.001), and IES-R score (*p* < 0.001). Marital status (*p* = 0.920), gender (female, *p* = 0.655), pre-morbid employment (*p* = 0.131), and TBSA (*p* = 0.577) were not found to be significant predictors. All significant variables were included for consideration in the model. Although not itself significant, gender was forced into the model due to its previously reported association with psychiatric outcomes in burn patients (14–16).

**Table 3:** **Univariate analysis of pain outcome with multiple covariates**.

Predictor	β coefficient	95% CI	Unadjusted *p* value^a^
Age	0.08	0.02–0.15	0.009
Female	−0.22	−1.17 to 0.74	0.655
Marital status	0.02	−0.34 to 0.38	0.920
Burn	1.17	0.27–2.07	0.012
Total body surface area %	−0.01	−0.04 to 0.02	0.577
Graft	−1.67	−2.84 to −0.49	0.006
BDI	0.09	0.06–0.13	<0.001
IES-R	0.04	0.02–0.05	<0.001
Pre-morbid employment	0.54	−0.16 to 1.24	0.131
Return to same employment	2.08	1.06–3.10	<0.001
Time off from work	0.51	0.20–0.82	0.001

*^a^Statistically significant defined as *p* ≤ 0.05*.

*BDI, Beck depression inventory*.

*IES-R, impact event scale – revised*.

Table 4 displays the results of the stepwise multiple regression. Amongst the eight variables identified by the univariate analyses, five variables were eliminated through the model building process. The final model (Model 5) yielded the following significant predictors: age (*p* = 0.012), graft (*p* = 0.009), and BDI (*p* < 0.001). Similar data variability of 36–41% was seen between models; the final model produced an *R*^2^ of 0.38, indicative of its ability to predict 38% of the variability amongst pain scores. No confounders were identified for the three variables that remained significant in the final model.

**Table 4:** **Stepwise multiple regression analysis examining predictors of pain**.

Significant predictors	Model 1			Model 2			Model 3			Model 4			Model 5		
	β coefficient	95% CI	*p* Value^a^	β coefficient	95% CI	*p* Value^a^	β coefficient	95% CI	*p* Value^a^	β coefficient	95% CI	*p* Value^a^	β coefficient	95% CI	*p* Value^a^
Age	0.09	0.02–0.16	0.016	0.09	0.02–0.16	0.014	0.07	0.01–0.14	0.023	0.08	0.01–0.14	0.020	0.08	0.02–0.14	0.012
Female	−0.62	−1.52 to 0.28	0.174	−0.60	−1.49 to 0.30	0.187	−0.66	−1.49 to 0.18	0.123	−0.61	−1.43 to 0.22	0.149	−0.57	−1.39 to 0.26	0.173
Burn	0.41	−0.88 to 1.70	0.527												
Graft	−1.54	−2.97 to −0.10	0.037	−1.57	−3.00 to −0.15	0.031	−1.33	−2.51 to −0.15	0.028	−1.21	−2.36 to −0.06	0.039	−1.43	−2.49 to −0.37	0.009
BDI	0.03	−0.04 to 0.10	0.439	0.03	−0.04 to 0.10	0.445	0.05	−0.01 to 0.12	0.093	0.08	0.04–0.12	< 0.001	0.08	0.04–0.12	< 0.001
IES-R	0.02	−0.01 to 0.05	0.271	0.02	−0.01 to 0.05	0.252	0.01	−0.02 to 0.04	0.379						
Time off work	−0.19	−0.76 to 0.38	0.511	−0.07	−0.51 to 0.36	0.743									
Return to same employment	0.80	−0.82 to 2.42	0.329	0.62	−0.89 to 2.13	0.417	0.56	−0.65 to 1.78	0.359	0.47	−0.73 to 1.66	0.438			
	Model 1 (*R*^2^ = 0.37)			Model 2 (*R*^2^ = 0.36)			Model 3 (*R*^2^ = 0.41)			Model 4 (*R*^2^ = 0.40)			Model 5 (*R*^2^ = 0.38)	

*^a^Statistically significant defined as *p* ≤ 0.05*.

*BDI, Beck depression inventory*.

*IES-R, impact event scale – revised*.

In order to test if there was a synergistic effect between predictors, paired and higher order interaction terms were forced into the model. Table 5 shows the results of the paired interactions for age and BDI, age and graft, and BDI and graft. A trend toward significance was noted for the interaction between age and depression (*p* = 0.052), as well as age and graft (*p* = 0.072). Similarly, there was found to be a trend toward significance of *p* = 0.057 for the three-way interaction between age, graft, and depression. Only the interaction between graft and depression was shown to be highly significant (*p* = 0.003) relative to VAS pain score.

**Table 5:** **Interaction analysis between pairs of significant predictors of pain**.

Significant predictors^a^	β coefficient	95% CI	*p* Value^b^
**INTERACTION 1. AGE AND BDI^c^**			
Age	0.01	−0.08 to 0.10	0.860
Female	− 0.44	−1.26 to 0.37	0.281
Graft	− 1.27	−2.32 to −0.22	0.018
BDI	− 0.11	−0.31 to 0.08	0.261
Interaction term (age and BDI^c^)	0.01	0.00–0.01	0.052
Interaction 1 (*R*^2^ = 0.41)			
**INTERACTION 2. AGE AND GRAFT**			
Age	0.21	0.05–0.37	0.009
Female	− 0.56	−1.37 to −0.25	0.175
Graft	3.31	−1.97 to 8.58	0.215
BDI	0.07	0.04–0.11	<0.001
Interaction term (age and graft)	− 0.15	−0.32 to 0.01	0.072
Interaction 2 (*R*^2^ = 0.41)
**INTERACTION 3. BDI^c^ AND GRAFT**			
Age	0.07	0.01–0.12	0.028
Female	− 0.37	−1.16 to 0.42	0.350
Graft	0.39	−1.17 to 1.95	0.619
BDI	0.19	0.11–0.27	<0.001
Interaction term (BDI^c^ and graft)	− 0.13	−0.22 to −0.04	0.003
Interaction 3 (*R*^2^ = 0.45)

*^a^The same predictors from Table 4, Model 5 were adopted to build this model*.

*^b^Statistically significant defined as *p* ≤ 0.05*.

*^c^BDI, Beck depression inventory*.

## Discussion

This survey of 104 survivors of The Station nightclub fire provides a unique opportunity to study chronic pain in individuals exposed to the same traumatic event. The findings suggest that pain in these subjects may be related to age, graft, and depressive symptoms. In addition, there was observed to be an interaction between graft and BDI score. The final model was able to explain 38% of the variability among pain scores.

### Burn injury and pain

This study found that fire survivors who sustained physical burn injury were more likely to report pain 28–56 months after the event, compared to those without burn. Burn size, as measured by total body surface area, was not a significant predictor of pain. Survivors who received grafts reported higher VAS pain scores than burn patients without grafts.

One possible explanation for the relationship between pain and grafting may be related to the physiology of the burn injury itself. The size of a burn alone is an unreliable indicator of clinical burn severity, since it is not uncommon for a given injury to contain regions of different depths. The requirement for skin grafting, however, may more accurately approximate deeper or more complex injury (17–19). The deeper the burn, the more progressive the structural damage to the dermis and underlying tissues. Deeper injuries heal more slowly, are more difficult to treat, and are more likely to be associated with local and systemic complications (20).

In deep burn injuries, all types of nociceptor afferents are affected, including both large-diameter, small myelinated, and unmyelinated C-fibers (3, 8). In grafted tissue, there may be preferential loss of large fibers (3), with deeper C-fibers capable of responding to pain to varying degrees (21). Damage to tissue surrounding a burn, either due to natural wound progression or as a consequence of medical intervention, may also result in peripheral nerve damage (3, 22). As neuronal cell regeneration occurs, an altered chemical milieu further contributes to abnormal excitability (3, 21).

Persistent, abnormal stimulus from the periphery contributes to changes in the central processing of pain and sensation (5, 6). These alterations may be transient in nature; Volz et al. recently demonstrated that, when one hand is exposed to increased sensory stimulation, pain threshold increases on the side of stimulation and decreases in the contralateral, unprovoked hand (23). Changes in peripheral and central sensory processing may lead to cortical reorganization and structural changes (3, 8, 21, 24), factors which have been linked to the development of chronic pain (4–6). Transcranial magnetic stimulation studies have shown subjects with neuropathic pain to have defective inhibition of the primary motor cortex (25, 26).

Studies of burn survivors have suggested that the co-occurrence of painful sensations and impaired sensibility are not uncommon, specially after deep burns and grafted injuries (1, 3, 8, 27). In one study of 121 burn survivors, patients with deep burns requiring skin grafts demonstrated decreased sensory perception by displaying significantly higher thresholds for pressure, discriminative, thermal, and pain modalities compared to individuals with superficial injury. Interestingly, sensory abnormalities were also seen in uninjured areas (8). Earlier, qualitative studies have noted similar findings. In a retrospective review of 60 grafted burn survivors, 97% of patients exhibited markedly diminished or absent response to various types of stimuli. Depth of burn was the best predictor of altered sensation, and non-grafted skin areas frequently recovered normal sensation (27). In multiple investigations, patients frequently described subjective, neuropathic-like symptoms of pain, paresthesias, and pruritus up to 12 years post-injury (1–3, 9, 21, 27, 28).

More recent studies have begun to elucidate the central neuropathic mechanisms that may contribute to chronic pain after burn. In one animal model, unilateral partial-thickness burn injury was noted to produce persistent bilateral allodynia associated with neuronal hyperexcitability and microglial activation in the spinal cord dorsal horn (24). Clinical studies have provided similar evidence of maladaptive neuroplasticity. Burn patients receiving transcranial direct current stimulation have been shown to demonstrate an overall decrease in cortical excitability characterized by an increase in intracortical inhibition and a decrease in intracortical facilitation and motor evoked potentials (29). Future research is needed to help correlate observed chronic pain phenomenon in fire survivors with pathophysiological changes.

### Age and pain

Age was found to be a significant predictor of pain among fire survivors. Despite the relatively young age range of the cohort, older age corresponded to higher pain scores. This is one of the first studies of pain in fire survivors to report such an association. In part, this may be due to the fact that many previous studies of pain and cutaneous sensibility following burn have been qualitative in nature and may not have had adequate statistical power to detect an age-related effect.

Several physical factors may predispose older individuals to develop a greater degree of pain following burn injury. Dermatologic changes that occur with aging include decreased cutaneous perfusion, alterations in skin strata thickness, changes in collagen and elastin distribution and quality, and reduced lipid content (30, 31). Age-related changes in proliferative response and inflammatory mediators may result in increased susceptibility to injury as well as a decline in wound repair (32, 33). In particular, older adults tend to suffer more severe burns at lower temperatures and in less time than their younger adult counterparts (33, 34).

Experimental data on age-related changes in pain perception have produced varied results. Some studies have shown that older individuals have enhanced pain sensitivity related to activation of pressure nociceptors in deep tissue (35–38). Other data have suggested age-related deterioration of endogenous pain inhibitory systems (39, 40). More recently, a comprehensive multimodal sensory approach was used to evaluate age-related pain sensitivity in a group of 40 individuals; results demonstrated a markedly increased temporal summation of heat pain in elderly individuals (38, 41). Temporal summation has been shown to be a centrally mediated process, thought to play an important role in pain amplification and the development of chronic pain conditions (38, 42, 43).

While physical factors and differences in pain perception are certainly important factors in chronic pain development with age, their relevance is less apparent for this study sample. At the time of the fire, survivors ranged in age from 19 to 57 years, representing a relatively youthful population. The study design also attempted to control the variable age for TBSA or the presence of graft; thus, it is less likely that burn injury itself would be the mechanism to explain a greater degree of pain in older individuals. Additional investigations are needed to evaluate factors that contribute to age-related chronic pain in fire survivors with and without physical injury.

### Psychological stressors and pain

This study demonstrated an association between depressive symptoms and pain scores among survivors of a large fire. This association was more pronounced for individuals who underwent grafting. Although many survivors reported symptoms of post-traumatic stress, such symptoms were not found to be a significant predictor of pain when controlled for other variables.

Chronic pain is a complex human experience that encompasses physical, psychological, and sociobehavioral components. It is well-recognized that individuals with chronic pain have an increased incidence of anxiety and mood disorders (44–47). Although existing data are scarce regarding emotional disturbance in fire survivors without physical injury, it is not uncommon for burn survivors to report psychological symptoms. Studies have demonstrated prevalence rates of 7–46% for depression and 9–45% for PTSD up to 1 year following burn injury (11, 48). In a retrospective study of 492 burn patients, symptom severity was significantly increased in those individuals who reported persistent pain up to 11 years after their injury. These patients were also found to recall higher levels of pain at rest, with dressing changes, and with physical activity during their acute care stay (48).

Recent experimental models may offer some explanation for the association between emotional trauma and chronic pain phenomena. Functional MRI has shown that, following depressed mood induction, brain responses to noxious thermal stimuli are increased in multiple areas including the inferior frontal gyrus and amygdala (49). Other studies have demonstrated a significant correlation between catastrophizing thoughts and changes in motor cortex excitability (7). In patients with burn injury, catastrophizing thoughts and the development of mood disturbance may result in dysregulation of the neural circuitry underlying emotion regulation; this could alter central pain processing and result in an amplification of pain perception (49, 50).

Although this study suggests a relationship between depression and chronic pain in fire survivors, the directionality of this relationship remains unclear. While not all survivors suffered burn injury, future investigation is needed to determine whether trauma-induced depression leads to pain amplification, or burn-related neuropathic pain leads to depressive symptoms.

### Study limitations and future considerations

A few limitations to the study are worth noting. Since only 104 of the estimated 330 survivors completed the survey, there is potential for a selection bias. There is also a risk of a reporting bias with the use of a self-reported questionnaire. Since subjects completed the questionnaire at different points in time, a comparison of long-term outcomes becomes more challenging. Furthermore, the cross-sectional study design precludes the evaluation of longitudinal change and the determination of causal relationships. Additional limitations in study design are presented in Schneider et al. (12).

While this study considered chronic pain as a function of VAS score, it may be more instructive to consider additional pain parameters, including location and sensory abnormalities. Other patient factors including pre-morbid pain and psychiatric characteristics, as well as pharmacologic intervention might also be considered. Since this was a self-reported questionnaire, duration and frequency of medication could not be appropriately collected.

This study highlights the importance of long-term follow-up of fire survivors, including those without burn injury. Although factors such as age and graft are non-modifiable, it is important to take into account that prompt intervention of depressive symptoms and post-traumatic stress could modify pain perception in this patient cohort. Understanding factors that contribute to chronic pain will help medical practitioners better care for their patients in the future.

## Conflict of Interest Statement

The authors declare that the research was conducted in the absence of any commercial or financial relationships that could be construed as a potential conflict of interest.
